# Increased Lipogenesis Is Important for Hexavalent Chromium-Transformed Lung Cells and Xenograft Tumor Growth

**DOI:** 10.3390/ijms242317060

**Published:** 2023-12-02

**Authors:** James T. F. Wise, Kazuya Kondo

**Affiliations:** 1 Wise Laboratory of Nutritional Toxicology and Metabolism, School of Nutrition and Food Sciences, College of Agriculture, Louisiana State University, 269 Knapp Hall, Baton Rouge, LA 70803, USA; 2 School of Nutrition and Food Sciences, College of Agriculture, Louisiana State University, Baton Rouge, LA 70803, USA; 3 School of Nutrition and Food Sciences, Louisiana State University Agriculture Center, Baton Rouge, LA 70803, USA; 4 Division of Nutritional Sciences, Pharmacology and Nutritional Sciences, College of Medicine, University of Kentucky, Lexington, KY 40536, USA; 5 Department of Oncological Medical Services, Graduate School of Biomedical Sciences, Tokushima University Graduate School, Tokushima City 770-8509, Japan

**Keywords:** hexavalent chromium, lipogenesis, cancer metabolism, fatty acid synthase, human lung cells

## Abstract

Hexavalent chromium, Cr(VI), is a known carcinogen and environmental health concern. It has been established that reactive oxygen species, genomic instability, and DNA damage repair deficiency are important contributors to the Cr(VI)-induced carcinogenesis mechanism. However, some hallmarks of cancer remain under-researched regarding the mechanism behind Cr(VI)-induced carcinogenesis. Increased lipogenesis is important to carcinogenesis and tumorigenesis in multiple types of cancers, yet the role increased lipogenesis has in Cr(VI) carcinogenesis is unclear. We report here that Cr(VI)-induced transformation of three human lung cell lines (BEAS-2B, BEP2D, and WTHBF-6) resulted in increased lipogenesis (palmitic acid levels), and Cr(VI)-transformed cells had an increased expression of key lipogenesis proteins (ATP citrate lyase [ACLY], acetyl-CoA carboxylase [ACC1], and fatty acid synthase [FASN]). We also determined that the Cr(VI)-transformed cells did not exhibit an increase in fatty acid oxidation or lipid droplets compared to their passage-matched control cells. Additionally, we observed increases in ACLY, ACC1, and FASN in lung tumor tissue compared with normal-adjacent lung tissue (in chromate workers that died of chromate-induced tumors). Next, using a known FASN inhibitor (C75), we treated Cr(VI)-transformed BEAS-2B with this inhibitor and measured cell growth, FASN protein expression, and growth in soft agar. We observed that FASN inhibition results in a decreased protein expression, decreased cell growth, and the inhibition of colony growth in soft agar. Next, using shRNA to knock down the FASN protein in Cr(VI)-transformed BEAS-2B cells, we saw a decrease in FASN protein expression and a loss of the xenograft tumor development of Cr(VI)-transformed BEAS-2B cells. These results demonstrate that FASN is important for Cr(VI)-transformed cell growth and cancer properties. In conclusion, these data show that Cr(VI)-transformation in vitro caused an increase in lipogenesis, and that this increase is vital for Cr(VI)-transformed cells.

## 1. Introduction

Hexavalent chromium (Cr(VI)) is a known carcinogen and exposure to it is associated with respiratory cancer [1,2,3,4]. Epidemiological studies of Cr(VI)-exposed chromate workers has shown they have an up to a 2 to 80-fold increased risk of developing lung cancers [2]. The exact mechanism of chromate-induced lung cancer remains elusive, but there are multiple potential mechanisms reported in the literature, including: (1) Cr(VI) is a potent genotoxic agent that acts as a carcinogen by inducing DNA double-strand breaks, chromosome instability, altered DNA repair, and genomic alterations; (2) Cr(VI) induces reactive oxygen species that cause downstream changes in protein signaling and redox imbalance; (3) Cr(VI) can alter oncogenes including c-Myc and epidermal growth factor receptor (EGFR) expression; and (4) Cr(VI) induces epigenetic modifications [3,4,5,6,7,8]. Growing evidence suggests that Cr(VI) is able to induce altered cellular energetics [5,9,10,11,12]. Our recent studies have shown Cr(VI) xenograft-derived tumor cells have mitochondrial respiration dysfunction [12]. Cr(VI) is also able to directly activate upstream factors that regulate de novo lipogenesis in human cells or alter the signaling patterns of these pathways (e.g., Nrf2, SREBP-1, and HIF-1α) [1,6,11,13].

Altered cellular energetics was first proposed to be a feature of cancer cells in the 1920s [14,15,16,17]. Growing evidence shows that cancer cells have increased glycolysis combined with an increase in lactic acid fermentation of pyruvates (anaerobic glycolysis). Conversely, normal cells have lower levels of glycolysis combined with low levels of lactic acid fermentation and high levels of pyruvate oxidation in their mitochondria (aerobic glycolysis). This change in the fate of the pyruvates after glycolysis persists even in the presence of physiological or ambient oxygen levels. The shift to anaerobic glycolysis in the presence of oxygen termed the “Warburg Effect,” is permanent [14,15,16,17]. Along with this change in the type of glycolysis in cancer cells, other energy pathways affected include mitochondrial respiration, one-carbon metabolism, and lipid metabolism [18,19]. These alterations in energy usage seem to be key to the survival of cancer cells and provide advantages in the tumor microenvironment [18,19,20,21]. It has also been reported that the energy-consuming processes of protein and DNA synthesis are increased in cancer cells. Lastly, the role of increased lipogenesis in cancer cells appears at least as important as increases in protein, DNA synthesis, and glycolysis [20,21]. These alterations in cellular energetics have recently been deemed a hallmark of cancer [18].

Most adult cells acquire necessary lipids via exogenous sources, like the adult’s diet, or they are synthesized in the liver and adipose tissue; thus, most tissues and cells have very low basal levels of lipid synthesis [20,21]. In many cancer systems, de novo lipogenesis proteins ATP-citrate lyase (ACLY), acetyl-CoA carboxylase 1 (ACC1), and fatty acid synthase (FASN) have been reported to be upregulated [20,21,22]. Specifically, increases in lipogenesis and these proteins have been observed in breast, colon, lung, ovarian, pancreatic, and prostate cancers [20,21,22,23,24,25,26]. The upregulation of these proteins is associated with poor prognoses of post-treatment survival in cancer patients [27,28]. Lipid increases in cancer cells are important for different aspects of cancer development: (1) they may be stimulated by oncogenic signaling (e.g., EGFR, HER2, HIF-1α, etc.); (2) they directly contribute to the growth and proliferation of cancer cells; (3) they protect against oxidative stress resistance and are involved in redox balance; (4) they provide advantages regarding cell survival under energy stress; and (5) they are involved with the invasive properties of cancer [20,22,29,30,31].

Furthermore, all three enzymes ACLY, ACC1, and FASN are rate-limiting enzymes involved in lipogenesis. In brief, ACLY converts citrate to acetyl-CoA, which is converted by ACC1 to malonyl-CoA. FASN then converts malonyl-CoA to palmitate [31]. In most normal adult human tissues, ACLY, ACC1, and FASN are expressed at minimal basal levels. Several clinical and basic studies have established the cellular and clinical significance of these lipid enzymes in cancer [32,33,34,35,36,37]. As noted above, lipogenesis is enhanced by these enzymes to provide selective growth and survival advantages to cancer cells. Pharmacological inhibitors of FASN inhibit lung cancer cell survival, induce programmed cell death, and reduce lung cancer xenograft tumor growth [38,39,40,41,42]. Some studies have also implicated these proteins in carcinogenesis, as their overexpression has been shown to be able to induce neoplastic transformation of skin and breast cell lines [43,44].

Little is known about the functions of lipogenesis in Cr(VI)-induced lung carcinogenesis. One report found that Cr(VI)-transformed cells and Cr(VI) tumor tissues had an increased ACLY expression [5]. In this study, we determined that lipogenesis plays a role in Cr(VI)-induced lung carcinogenesis and that Cr(VI)-transformed lung cells exhibit upregulated lipogenesis. This study also showed that an inhibition of FASN dramatically inhibits cell proliferation and loss of anchorage independence in Cr(VI)-transformed lung cells and xenograft tumor growth in nude mice.

## 2. Results

### 2.1. Chromium(VI)-Transformed BEAS-2B Cells Have Increased Lipogenesis and Related Proteins

**Figure 1:**
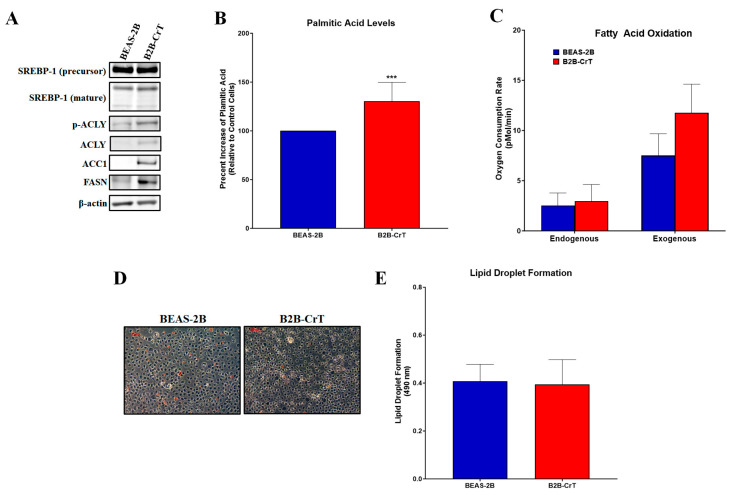
Chromium(VI)-transformed BEAS-2B cells have increased levels of lipogenesis and related proteins. (**A**) Cr(VI)-transformed BEAS-2B cells (B2B-CrT cells) had increased ACLY, pACLY, ACC1, and FASN protein expressions compared to passage-matched control BEAS-2B cells. (**B**) B2B-CrT cells had increased palmitic acid levels compared to passage-matched control BEAS-2B cells (data are presented as percent increase of passage-matched control BEAS-2B cells). (**C**,**D**) B2B-CrT cells had no increases in lipid droplet formation (Oil Red O staining) when compared to passage-matched control BEAS-2B cells. (**E**) Endogenous and exogenous fatty acid oxidation were measured using the Seahorse Analyzer. B2B-CrT cells did not have increased fatty acid oxidation when compared to their passage-matched control BEAS-2B cells. Data represent at least three independent experiments, error bars represent the SEM, *** *p <* 0.001 compared to passage-matched control cells. The scale bar is 100 µm.

Using the BEAS-B cells and Cr(VI)-transformed BEAS-2B cells (B2B-CrT) from our previous study, we investigated lipogenesis endpoints in these cells (protein and functional palmitic acid levels) [12]. The B2B-CrT cells had increased ACLY, pACLY, ACC1, and FASN protein expressions compared to passage-matched control BEAS-2B cells. SREBP-1 protein expression levels were not increased, indicating that the increase in protein expression was not due to increases in transcription (Figure 1A). B2B-CrT cells had increased palmitic acid levels compared to passage-matched control cells (Figure 1B), confirming a functional change in the lipogenesis pathway. Typically, an increase in lipid production is observed in cancer cells for various endpoints (e.g., membrane synthesis, changing lipid profiles, protection against oxidation, energy usage, and lipid droplet storage). We measured energy usage and lipid droplet storage. We saw no major changes in endogenous or exogenous fatty acid oxidation of the B2B-CrT cells compared to passage-matched control BEAS-2B cells (Figure 1C). Lastly, to measure lipid droplet formation, we employed Oil Red O staining; no changes in lipid droplet formation were observed in the Cr(VI)-transformed cells compared to passage-matched control cells (Figure 1D,E). 

### 2.2. Chromium(VI)-Transformed BEP2D Cells Have Increased Lipogenesis and Related Proteins

**Figure 2:**
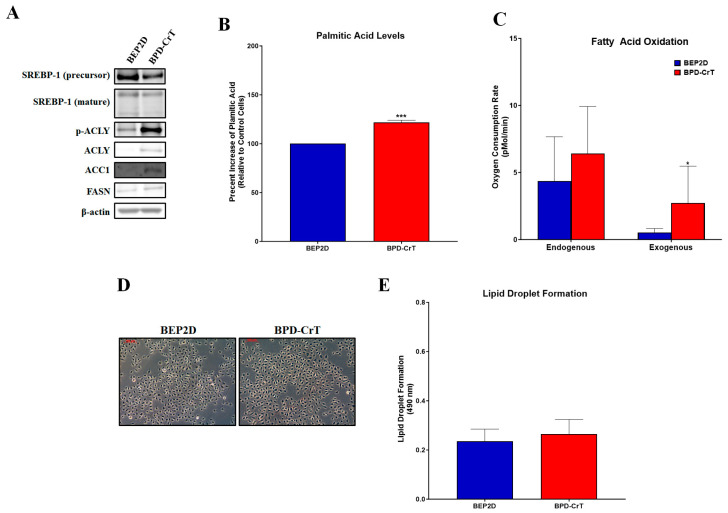
Chromium(VI)-transformed BEP2D cells have increased lipogenesis and related proteins. (**A**) Cr(VI)-transformed BEP2D cells (BPD-CrT cells) had increased ACLY, pACLY, ACC1, and FASN protein expressions compared to passage-matched control BEP2D cells. (**B**) B2B-CrT cells had increased palmitic acid levels compared to passage-matched control BEP2D cells (data presented as percent increase of passage-matched control BEP2D cells) (**C**,**D**) BPD-CrT cells had no increases in lipid droplet formation (Oil Red O staining) when compared to passage-matched control BEP2D cells. (**E**) Endogenous and exogenous fatty acid oxidation were measured using the Seahorse Analyzer. BPD-CrT cells did not have increased fatty acid oxidation when compared to their passage-matched control BEP2D cells. Data represent at least three independent experiments, error bars represent the SEM, * *p <* 0.05; *** *p <* 0.001 compared to passage-matched control cells. The scale bar is 100 µm.

We used BEP2D cells and Cr(VI)-transformed BEP2D Cells (BPD-CrT) from the Wise Laboratory at the University of Louisville, to investigate lipogenesis endpoints in these cells. We aimed to confirm that our results with the BEAS-2B cells were not cell line specific. The BPD-CrT cells had increased ACLY, pACLY, ACC1, and FASN protein expressions compared to passage-matched controls (Figure 2A). SREBP-1 protein expression levels were not increased, indicating that the increase in protein expression was not due to increased levels of transcription (Figure 2A). BPD-CrT cells had increased palmitic acid levels compared to passage-matched control cells (Figure 2B). We saw no changes in endogenous fatty acid oxidation of the BPD-CrT cells compared to passage-matched control BEP2D cells, However, exogenous fatty acid oxidation was increased, suggesting these cells may metabolize lipids at a faster rate (Figure 2C). Lastly, no changes in lipid droplet formation were observed in the Cr(VI)-transformed cells compared to passage-matched control cells (Figure 2D,E).

### 2.3. Chromium(VI)-Transformed WTHBF-6 Cells Have Increased Lipogenesis and Related Proteins

**Figure 3:**
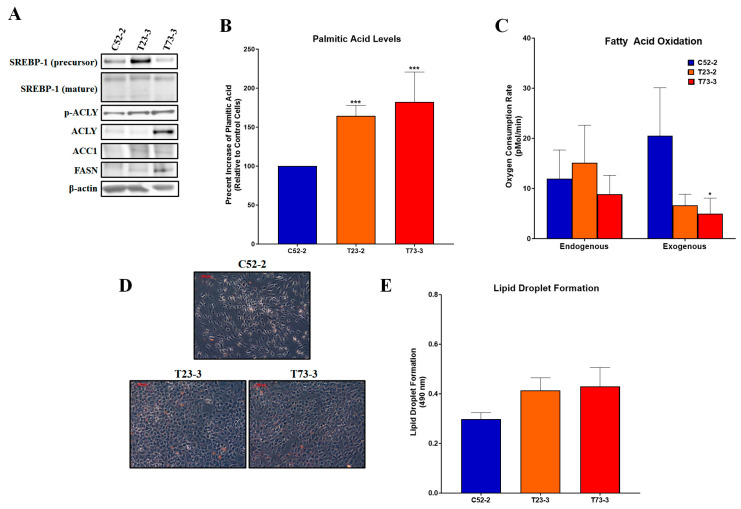
Chromium(VI)-transformed WTHBF-6 cells have increased lipogenesis and related proteins. (**A**) Cr(VI)-transformed WTHBF-6 cells (T23-3 and T73-3 cells) had increased ACLY, pACLY, ACC1, and FASN protein expressions compared to passage-matched control WTHBF-6 cells (C52-2). (**B**) T23-3 and T73-3 cells had increased palmitic acid levels compared to passage-matched control cells (data presented as percent increase of passage-matched control WTHBF-6 cells) (**C**,**D**) T23-3 and T73-3 cells had no increases in lipid droplet formation (Oil Red O staining) when compared to passage-matched control cells. (**E**) Endogenous and exogenous fatty acid oxidation were measured using the Seahorse Analyzer. T23-3 and T73-3 cells did not have increased fatty acid oxidation when compared to their passage-matched control cells. Data represent at least three independent experiments, error bars represent the SEM; * *p <* 0.05, *** *p <* 0.001 compared to passage-matched control cells. The scale bar is 100 µm.

Next, using WTHBF-6 cells (C52-2) and Cr(VI)-transformed WTHBF-6 cells (T23-3 and T73-3) from the Wise Laboratory at the University of Louisville, we investigated lipogenesis endpoints in these cells. T23-3 and T73-3 cells had increased ACLY, pACLY, ACC1, and FASN protein expressions compared to passage-matched controls (Figure 3A). Mature SREBP-1 protein expression levels were not increased, indicating that the increase in protein expression was not due to increased levels of transcription. The precursor SREBP-1 protein level was increased but its mature form is not involved with transcription of lipogenesis proteins (Figure 3A). T23-3 and T73-3 had increased palmitic acid levels compared to their passage-matched control cells (Figure 3B). We saw no changes in endogenous or exogenous fatty acid oxidation of the T23-3 and T73-3 cells compared to passage-matched control cells (Figure 3C). We observed no changes in lipid droplet formation in the Cr(VI)-transformed cells compared to passage-matched control cells (Figure 3D,E).

### 2.4. Increased FASN Expression Is Important for Chromium(VI)-Transformed Cell Survival

**Figure 4:**
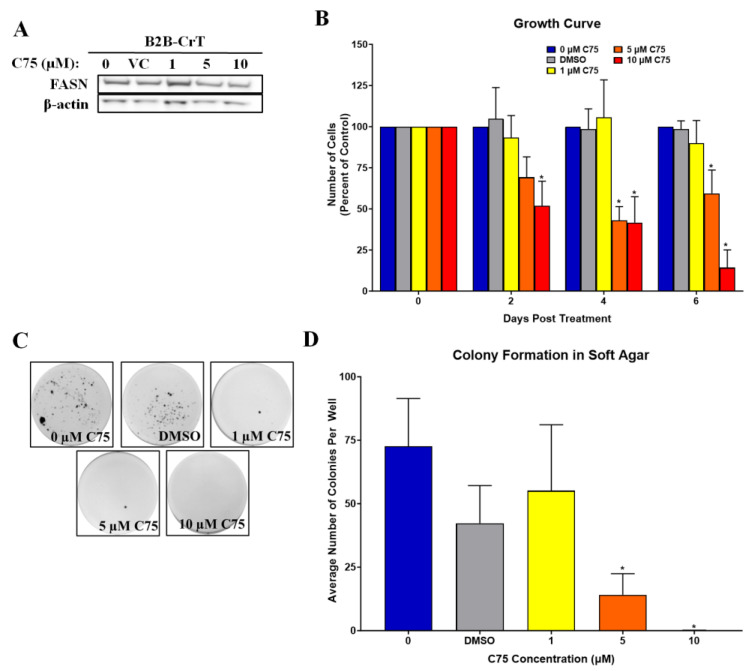
Drug inhibition of FASN in chromium(VI)- transformed cells results in decreased survival and growth. (**A**) 24 h treatment with 0, 1, 5, and 10 µM C75 (FASN inhibitor) decreased FASN protein expression in Cr(VI)-transformed BEAS-2B cells (B2B-CrT cells). (**B**) 24 h treatment with 0, 1, 5, and 10 µM C75. 5 and 10 µM C75 decreased cell growth of B2B-CrT cells (data presented as percent decrease of untreated B2B-CrT cells). (**C**,**D**) 24 h treatment with C75 decreased B2B-CrT soft agar colony formation. (**D**) 24 h treatment with C75 decreased B2B-CrT soft agar colony formation. DMSO was used as a vehicle control (VC). Data represent at least three independent experiments, error bars represent the SEM, * *p <* 0.05 compared to passage-matched control cells.

To determine if the increase in lipogenesis is important to Cr(VI)-transformed cell survival and cancer properties, we examined soft agar colony formation, proliferation, and tumor growth after drug inhibition (C75) of FASN. We chose to focus on the Cr(VI)-transformed BEAS-2B cells, since all three cell types showed the same metabolism changes following neoplastic transformation with chromium when doing FASN manipulations. Multiple reports have shown that inhibition of FASN leads to decreased cancer cell growth and tumor growth [23,26,41]. Following a 24 h treatment with 0, 1, 5, and 10 µM C75, B2B-CrT, C75 treated cells had decreased FASN expressions (Figure 4A). C75 treatment decreased palmitic acid levels in B2B-CrT cells (Figure 4B). Treatment with C75 also decreased the soft agar colony formation properties of B2B-CrT cells (Figure 4C,D).

### 2.5. Increased FASN Expression Is Important for Chromium(VI)-Transformed Cell Tumorigenesis

**Figure 5:**
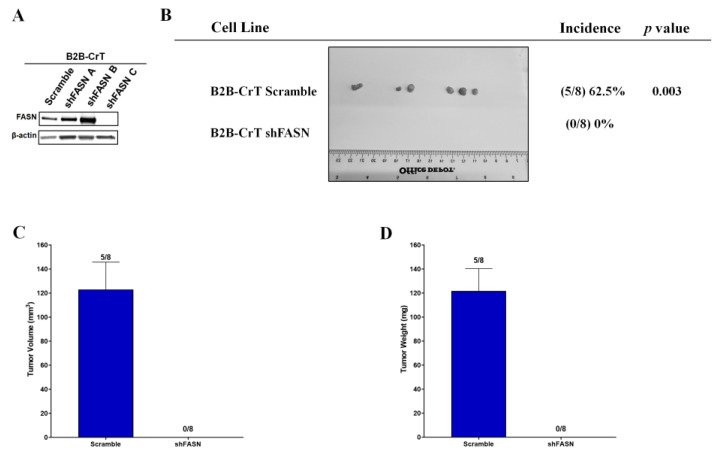
Increased FASN expression is important for Cr(VI)-transformed cells tumor growth. (**A**) shRNA inhibition of FASN results in decreased FASN protein expression in Cr(VI)-transformed BEAS-2B cells (B2B-CrT cells), scrambles and three shRNA sequences were used. (**B**) shRNA inhibition of FASN results in loss of tumor incidence in nude mice for B2B-CrT cells, *p <* 0.003. (**C**,**D**) Tumor size and weight of B2B-CrT Cells. There were eight injection sites per cell line and error bars represent the SEM.

To determine if the increase in FASN expression is important to Cr(VI)-transformed cell tumorigenesis, we examined the xenograft tumor development of Cr(VI)-transformed BEAS-2B cells following the loss of FASN expression using shRNA. We chose to focus on just the BEAS-2B cells, since all three cell types showed the same metabolism changes following neoplastic transformation with chromium and have all been shown to grow tumors in nude mice [12]. Following the shRNA inhibition, B2B-CrT had a loss of visible FASN expression with shFASN sequence C (Figure 5A). These cells and scramble sequences were used in the tumorigenesis study; B2B-CrT scramble cells grew tumors in five out of the eight injection sites and there were no tumors found in mice injected with B2B-CrT shFASN *p <* 0.003 (Figure 5B). Tumor volume and weight were measured in the B2B-CrT (Figure 5C,D).

### 2.6. Chromium(VI)-Induced Lung Tumors Have Increased Lipogenesis Protein Expressions

**Figure 6:**
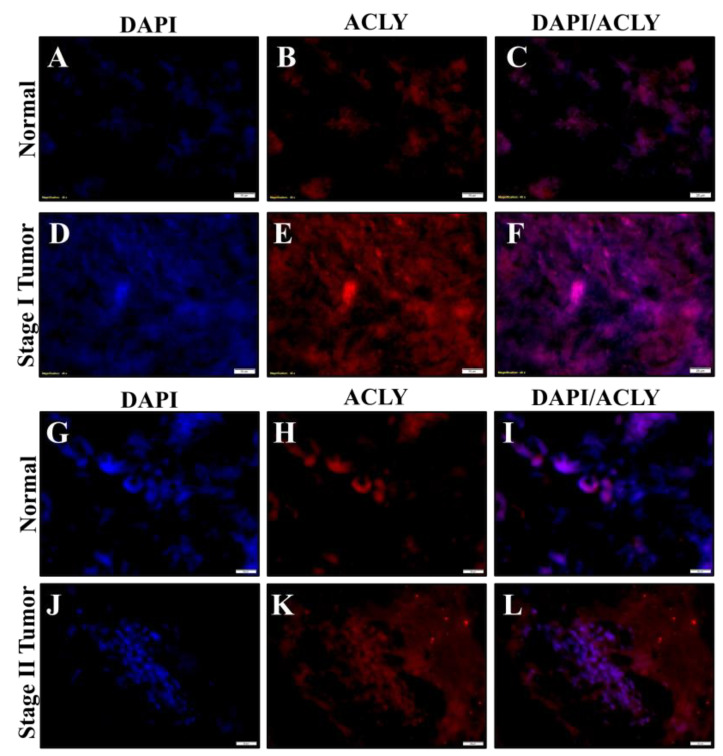
Chromate lung tumors show increased ACLY protein expression. Formalin-fixed normal lung tissue from lung tumor tissues and their adjacent normal tissues from workers diagnosed with stage I and II lung adenocarcinomas due to occupational exposure to Cr(VI) were subjected to immunofluorescence staining of their nuclei for examination of expressions of ACLY (red) and DAPI (blue). (**A**–**C**) DAPI and ACLY staining for the normal adjacent tissue of the stage I tumor. (**D**–**F**) DAPI and ACLY staining for the normal stage I tumor. (**G**–**I**) DAPI and ACLY staining for normal adjacent tissue of the stage II tumor. (**J**–**L**) DAPI and ACLY staining for the stage II tumor. The scale bar is 20 µm.

**Figure 7:**
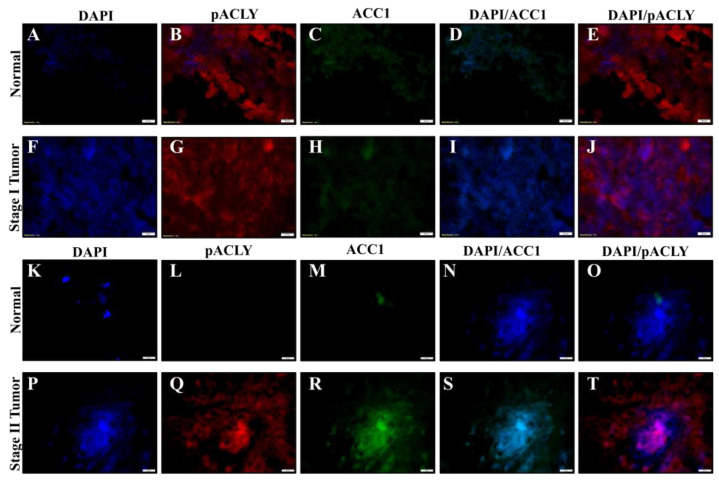
Chromate lung tumors show increased pACLY and ACC1 protein expressions. Formalin-fixed normal lung tissue from lung tumor tissues and their adjacent normal tissues from workers diagnosed with stage I and II lung adenocarcinomas due to occupational exposure to Cr(VI) were subjected to immunofluorescence staining of their nuclei for examination of expressions of p-ACLY (red), ACC1 (green), and DAPI (blue). (**A**–**E**) DAPI, pACLY, and ACC1 staining for the normal adjacent tissue of the stage I tumor. (**F**–**J**) DAPI, pACLY, and ACC1 staining for the stage I tumor. (**K**–**O**) A DAPI, pACLY, and ACC1 staining for the normal adjacent tissue of the stage II tumor. (**P**–**T**) DAPI, pACLY, and ACC1 staining for the stage II tumor. The scale bar is 20 µm.

**Figure 8:**
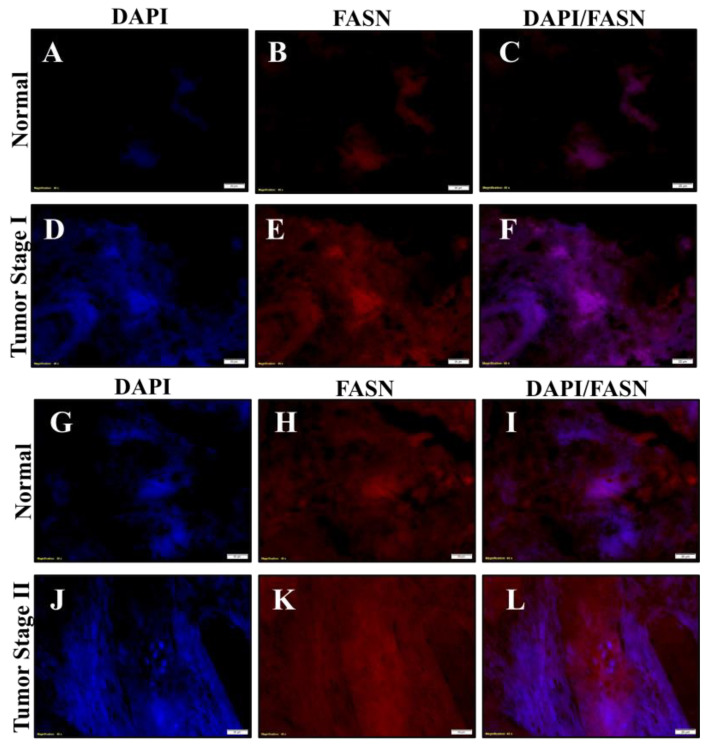
Chromate lung tumors show increased FASN protein expression. Formalin-fixed normal lung tissue from lung tumor tissues and their adjacent normal tissues from workers diagnosed with stage I and II lung adenocarcinomas due to occupational exposure to Cr(VI) were subjected to immunofluorescence staining of their nuclei for examination of expressions of FASN (red) and DAPI (blue). (**A**–**C**) DAPI and FASN staining for the normal adjacent tissue of the stage I tumor. (**D**–**F**) DAPI and FASN staining for the stage I tumor. (**G**–**I**) DAPI and FASN staining for normal adjacent tissue of the stage II tumor. (**J**–**L**) DAPI and FASN staining for the stage II tumor. The scale bar is 20 µm.

Formalin-fixed lung tissue slides from tumors and normal adjacent tissues obtained from workers exposed to Cr(VI) were used for fluorescence immunostaining. We stained stage I and stage II tumors for ACLY, pACLY, ACC1, and FASN. DAPI was used to stain the location of the nuclei. We then determined visual increases based on observations of the relative visual protein expression when merged with DAPI. The total ACLY expression was visually increased in the Cr(VI)-tumor lung tissue (Figure 6E,K) when compared to the adjacent normal lung tissue (Figure 6B,H). pACLY was visually increased in the Cr(VI)-tumor lung tissue (Figure 7G,Q) as compared to the adjacent normal lung tissue (Figure 7B,L); the visually observed increased in the expression of pACLY was clearer in the stage II tumor (Figure 7Q). ACC1 was visually increased in the Cr(VI)-tumor lung tissue (Figure 7H,R) as compared to the adjacent normal lung tissue (Figure 7C,M), and the visually observed increased in the expression of ACC1 was clearer in the stage II tumor (Figure 7R). FASN was visually increased in the Cr(VI)-tumor lung tissue (Figure 8E,K) as compared to the adjacent normal lung tissue (Figure 8B,H). These results confirm our cell culture findings and show that lipogenesis protein expressions are increased in Cr(VI)-induced lung tumor tissues.

## 3. Discussion

We report on the lipid metabolism changes in Cr(VI)-induced transformation. We saw that key lipogenesis proteins, namely ACLY, ACC1, and FASN, were increased in three sets of Cr(VI)-transformed lung cells. These data match the literature as they state that cancer cells have increased lipogenesis proteins and that Cr(VI)-transformed cells and Cr-tumors have increased ACLY expressions [5,20,22]. Previous studies have indicated that overexpression of these lipogenesis proteins is important to the carcinogenesis mechanism of Cr(VI). In human mammary epithelial cells, overexpression of FASN leads to neoplastic culture in vitro, and in human skin cells, overexpression of ACC1 drives the neoplastic transformation of these cells [43,44]. Further, overexpression of FASN in mammary epithelial cells drives neoplastic transformation and has been shown to be important to the carcinogenesis of breast cancer [44].

We demonstrated drug inhibition (C75) of FASN led to decreased cell growth, FASN protein expression, palmitic acid levels, and soft agar colony formation of Cr(VI)-transformed BEAS-2B cells. These results demonstrate that increased de novo lipogenesis is important for Cr(VI)-transformed cells and overexpression of FASN is a key protein that enhances the carcinogenic properties of these cells. It was reported that C75 inhibition in A549 cells inhibited FASN and cancer properties of these cells; our data match these results [41]. The data in this study also match those of reports on lung cancer where overexpression of these lipogenesis proteins is seen in lung cancer cell lines and patient tumors [22,23,24,25,27,28,41,45,46].

In human lung cancer patients, overexpression of ACLY and FASN in tumor tissues are observed and associated with a poor post-treatment survival rate [22,23,24,25,28]. Similarly, increased lung tumor expression p-ACC (phosphorylation of ACC1/ACC2 leads to inhibition) is linked to increased patient survival [45]. These studies demonstrated that the increases in lipogenesis in cell culture studies is translational in lung cancer patients. We stained tumor and adjacent normal tissue from the chromate-induced lung cancer tumors of two chromate workers. One subject was also a smoker. We observed that the total ACLY, pACLY, ACC1, and FASN expressions were increased in the Cr-tumor tissue as compared to the normal adjacent tissues. Likewise, we observed that p-ACC1 was increased in normal tissue as compared with tumor tissue (data not shown), while the total ACC1 was increased in tumor tissue compared to normal tissue. These data are important because they demonstrate that our cell culture results are consistent with human Cr-induced lung cancers.

From our FASN inhibition data it is not clear if the lack of growth in the growth curve experiments is due to cell death or cell cycle arrest; however, blocking overexpressed FASN leads to a cytotoxic buildup of malonyl-CoA, which then results in cell death [30]. Therefore, it is likely that the inhibition of FASN in Cr(VI)-transformed cells results in cell death by leading to the cytotoxic accumulation of malonyl-CoA. Furthermore, it has been demonstrated that FASN is important for lung cancer cells’ tumorigenesis [41]. We demonstrated by using shRNA knock-down of FASN that FASN is important for Cr(VI)-transformed cell tumorigenesis based on the loss of xenograft tumor growth in nude mice.

Our data demonstrate that Cr(VI)-transformed cells have increased lipogenesis-related proteins and increased lipogenesis (as measured with the palmitic acid levels). We did not observe that this increase in lipogenesis is for energy usage or storage (lipid droplet formation) but rather is involved with cell proliferation and tumor growth; further, these changes may be involved in other lipogenesis-related changes in these cells, which can be a research target for future studies. Additional future studies are needed to investigate the interactions between up-stream factors with the change in lipogenesis in Cr(VI)-transformed cells. Specifically, the role of AKT or EGFR in the lipogenesis changes in Cr(VI)-transformed cells needs to be investigated given that both AKT and EGFR can regulate metabolism [20]. Further, EGFR and FASN form a positive feedback loop in cancer cells, which needs to be investigated in Cr(VI)-transformed lung cells as it may provide more mechanistic evidence of the role of FASN in Cr(VI)-transformed cell growth [47,48].

## 4. Materials and Methods

### 4.1. Reagents

Sodium chromate, glutamine (for Seahorse tests), oligomycin, 2-deoxyglucose, rotenone, and antimycin A were purchased from MilliporeSigma (Burlington, MA, USA). Trypsin/EDTA, sodium pyruvate, penicillin/streptomycin, PBS (phosphate-buffered solution), LHC medias, and L-Glutamine were purchased from Thermo Fisher Scientific (Waltham, MA, USA). Dulbecco’s minimal essential medium and Ham’s F-12 (DMEM/F-12) were purchased from Mediatech (Herndon, VA, USA). Cosmic calf serum was purchased from Hyclone (Logan, UT, USA). Tissue culture dishes, flasks, and plasticware were purchased from Falcon Labware (Becton-Dickinson, Franklin Lakes, NJ, USA). XF base medium was purchased from Agilent Technologies (Santa Clara, CA, USA). The 4-Methylene-2-octyl-5-oxotetrahydrofuran-3-carboxylic acid (C75) and Oil Red O stain were purchased from Millipore Sigma (St. Louis, MO, USA). Formaldehyde was purchased from Ricca Chemical (Batesville, IN, USA).

### 4.2. Antibodies

Primary antibodies for α-tubulin (CST3873) acetyl-CoA carboxylase 1 (CST4190), ATP-citrate lyase (CST4332), fatty acid synthase (CST3189), phosphorylated acetyl-CoA carboxylase 1 (CST3661), and phosphorylated ATP-citrate lyase (CST4331) were purchased from Cell Signaling (Danvers, MA, USA). The primary antibody for sterol regulatory binding protein 1 (BD557306) was purchased from BD Biosciences (Franklin Lakes, NJ, USA). The primary antibody for β-actin (8A5441) was purchased from Sigma-Aldrich (St. Louis, MO, USA). For the Western blot test, primary antibodies were diluted at 1:1000 (except β-actin, which was diluted at 1:5000) in 1% BSA in TBST with 200 μL sodium azide. For the immunofluorescence antibodies, primary antibodies against ACLY, ACC1 (as known as ACCα), and FASN were purchased from Abcam (Cambridge, UK) and primary antibodies against p-ACLY was purchased from Santa Cruz Biotechnology (Santa Cruz, CA, USA), and secondary antibodies for anti-rabbit and anti-mouse were purchased from Cell Signaling (Danvers, MA, USA). Secondary antibodies were diluted in 2.5% buffer (BSA), at 1:5000 (β-actin was 1:10,000). For immunofluorescence, primary antibodies were diluted at 1:100 in 1% BSA in TBS and secondary antibodies were diluted in TBST. Secondary antibodies for anti-mouse and anti-rabbit and anti-mouse IgG (diluted to 1:100) were purchased from Invitrogen (Carlsbad, CA, USA).

### 4.3. Cell Culture

BEAS-2B, BEP2D, and WTHBF-6 were used as model human lung cells. BEAS-2B comprise SV40 large T-immortalized human bronchial airway cells purchased from ATCC [49]. BEP2D cells are HPV-immortalized human bronchial epithelial cells, which were received as a gift from Dr. Curtis Harris at NIH [50]. WTHBF-6 cells are hTERT-expressing human lung fibroblasts and were received as a gift from the laboratory of Dr. John Pierce Wise, Sr. at the University of Louisville, KY [7]. WTHBF-6 and Cr(VI)-transformed WTHBF-6 cell lines were cultured in a 50:50 mix of Dulbecco’s minimal essential medium and Ham’s F12 medium plus 15% cosmic calf serum, 1% L-glutamine, and 1% penicillin/streptomycin. Both the BEAS-2B and Cr(VI)-transformed BEAS-2B cell lines were cultured in LHC-9 media. Both the BEP2D and Cr(VI)-transformed BEP2D cell lines were cultured in LHC-8 media. All cells were screened for growth in soft agar and in nude mice to confirm the Cr(VI)-transformed cells and passage-matched control cells; this confirmed that the passage-matched control cells did not spontaneously transform [12]. Additionally, karyotypes were screened by Dr. John P. Wise, Sr. at the University of Louisville, KY to authenticate the passage-matched control cell lines. All cells were maintained in a 37 °C humidified incubator with 5% CO_2_. Cells were sub-cultured at least once a week using a 0.25% trypsin/1 mM EDTA solution and all experiments were performed on logarithmically growing cells.

### 4.4. Chromium(VI)-Transformed Cells

The Cr(VI)-transformed BEAS-2B cells we used are described in a previous report [12]. Both the Cr(VI)-transformed BEP2D and WTHBF-6 cells were received as gifts from the laboratory of Dr. John P. Wise, Sr. at the University of Louisville (Louisville, KY, USA) [4,7].

### 4.5. Seahorse Extracellular Flux Analysis

The Seahorse XF96 Extracellular Flux Analyzer (Agilent Technologies, Santa Clara, CA, USA) was used to measure fatty acid oxidation in all cells. At 24 h before the assays, the BEAS-2B, BEP2D, and WTHBF-6 cells were seeded at densities of 4.0 × 10^4^, 4.5 × 10^4^, and 3.5 × 10^4^ cells per well in an XF96 plate, respectively. The seeding densities used for the Cr(VI)-transformed cells were the same as their passage-matched control cells. To measure the rate of fatty acid oxidation, the cells were incubated overnight in their normal cell culture media with a 0.5 mM L-carnitine supplementation in XF96 cell culture plates. Forty-five minutes before the beginning of the oxygen consumption rate (OCR) measurement, the cells were switched to the assay medium (DMEM with 25 mM glucose, 0.5 mM carnitine, 2 mM glutamine, and 1 mM pyruvate). Etomoxir (40 µM) was added to one set of the cells to reveal the amount of FAO-associated OCR. The endogenous FAO respiration was calculated by subtracting the OCR levels in ETO-treated cells receiving BSA from those in the untreated cells receiving BSA. The exogenous FAO respiration was calculated by subtracting the OCR levels in ETO-treated cells receiving BSA-palmitate from those in the untreated cells receiving BSA-palmitate. All Seahorse experiments were performed using at least three independent experiments in the Redox Metabolism Shared Resource Facility at the University of Kentucky.

### 4.6. Soft Agar Assay

Soft agar colony formation assays were performed as described [12]. Briefly, 2 mL of 0.67% agar in LHC-9 media was placed into each well of a 6-well culture plate. A suspension (2 mL) containing 5 × 10^4^ cells was mixed with 2 mL of 0.33% agar-LHC-9 placed on the previous bottom layer of the agar and grown for 8 weeks. After plating (24 h later), cultures were examined microscopically to confirm the absence of large clumps of cells. Colonies were stained with 5% 4-nitro blue tetrazolium chloride for 24 h. Three independent experiments were performed.

### 4.7. Growth Curve Analysis

Cr(VI)-transformed BEAS-2B cells (B2B-CrT cells) were treated with the FASN inhibitor C75 for 24 h in a 100 mm dish. Cells were washed with PBS once, trypsinized, and centrifuged at 1000 rpm at 4 °C for 5 min, and then resuspended in media after collecting the cell pellet. Cells were counted with the Moxi GO II (ORFLO Technologies, Ketchum, ID). Each concentration was seeded into 3 wells of a 6-well plate at 12,500 cells (this was considered day 0). One well per concentration was harvested and counted every two days for up to six days. Data were then presented as percentages of the untreated control cells. Three independent experiments were carried out.

### 4.8. Drug Compound Treatments

C75 was prepared in DMSO of a 10 mM concentration and treated in 10 mL of fresh media at various concentrations as described in the results, as indicated. DMSO was used as a vehicle treatment.

### 4.9. Palmitic Acid Levels

To measure palmitic acid (palmitate) levels, 1 × 10^6^ cells were harvested. Cells were homogenized by pipetting in 200 μL of chloroform with 1% triton-x. The samples were then centrifuged at 16× *g* for 5 min at 4 °C and the lower (non-polar) phase was transferred to a new tube and dried at 50 °C. Then, the samples were vacuum centrifuged for 30 min and resuspended in 200 μL of sample buffer; 50 µL of sample buffer was used per well. Then, the manufacturer’s instructions for the Free Fatty Acid Quantification Kit from BioVision (Milpitas, CA, USA) were followed and quantification was performed at 560 nm on the BioTek EL800 plate reader (Winooski, VT, USA). Using a standard curve and correcting for the ¼ dilution factor, palmitic acid levels were determined for the samples. Three independent experiments were performed for each cell line.

### 4.10. Protein Preparation and Quantification

Cells were washed with cold (4 °C) PBS 2× and scraped with a cell scraper on ice in cold PBS. Pellets were collected in a microcentrifuge tube and centrifuged at 5000× *g* at 4 °C for 5 min. The PBS was then aspirated. Pellets were homogenized in 60–100 μL of RIPA buffer (containing protease inhibitors and phosphatase inhibitors). Pellets were then incubated on ice for 10 min and then sonicated. Pellets were centrifuged at 16× *g* for 10 min at 4 °C. Then, a Bradford assay was performed to measure the protein concentrations. Samples were prepared at 30 μg/15 μL in RIPA buffer with sample buffer (glycerol, 2-mercaptoethanol, SDS, Tris-HCL buffer (pH 6.8), and bromophenol blue).

### 4.11. Oil Red O Staining

Cells were seeded into 6-well plates and when the cells reached 80–90% confluence they were processed for lipid droplet formation. Briefly, the dishes were washed with PBS three times and then fixed with 4% formaldehyde for 40 min. Cells were then incubated in Oil Red O solution (3 parts Oil Red O to 2 parts water) for 40 min. Following staining with Oil Red O, the dishes were washed 3 times with water and imaged on the Zeiss Observer A1 Microscope (Carl Zeiss AG, Germany). Following imaging, the cells were dissolved and quantified. Quantification was carried out at 490 nm on the BioTek EL800 plate reader (Winooski, VT, USA). Three independent experiments were performed

### 4.12. Western Blot Analysis

Western blot analysis was carried out using standard laboratory procedures. Briefly, the protein samples were heated at 95 °C for 10 min and then centrifuged at 16× *g* for 10 min at 4 °C. Protein samples (30 µg) were then loaded into mini-prep 3–8% tris-acetate gels and run through a tris-acetate buffer. Running time was about 90 min at −125 v. Gels were then transferred to nitrocellulose membranes overnight, at 20 mAmps. To visualize and confirm this transfer, the membranes were incubated in ponceau S for 5 min. Membranes were then blocked in a 5% blocking buffer (BSA) for 1 h. Membranes were incubated in primary antibodies for 72 h. Following primary antibody incubation, the membranes were washed 6 times with 1x TBST buffer over 1 h. Membranes were incubated with secondary antibodies (HRP antibodies) for 1 h in a 2.5% blocking buffer (BSA in TBST). Following incubation with secondary antibodies, the membranes were washed 6 times with 1x TBST buffer over 1 h. Membranes were then exposed to ECL reagent for up to 5 min and developed on the Azure c600 (Azure Biosystems, Dublin, CA, USA). We then observed visual changes in the protein expression to determine whether increases or decreases, or no changes, occurred.

### 4.13. shRNA Transfection

Lentivirus samples containing different shRNA for FASN (pGFP-C-shLenti) were purchased from OriGene Technologies (Rockville, MD, USA), catalog no. TL313058V. Cr(VI)-transformed BEAS-2B cells underwent transduction following the manufacturer’s instructions. Briefly, transduction was performed in antibiotic-free media with 10 µg/mL polybrene overnight with either the scramble sequence shRNA or FASN shRNA A, B, or C. Then, 72 h after transduction, the cells were given a puromycin (antibiotic selection) for up to 3 weeks and screened for a loss of FASN using Western blot.

### 4.14. Xenograft Studies

The Xenograft tumor studies were conducted in accordance with NIH animal use guidelines, and the experimental protocol was approved by the Institutional Animal Care and Use Committee of the University of Kentucky at Lexington. Male athymic nude mice (NU/NU, 6–8 weeks old; The Jackson Laboratory, Bar Harbor, ME, USA) were housed in a pathogen-free room in the animal facilities at the Chandler Medical Center, University of Kentucky. Cells (~1 × 106 cells per mouse) from each cell line were re-suspended in serum-free medium with basement membrane matrix (BD Biosciences) and subcutaneously injected into the flanks of nude mice and allowed to grow for up to 6 months (8 injection sites per cell line), as previously described [12]. At the end of the experiment, the mice were sacrificed, and the tumor weight, tumor incidence, and tumor volume were determined as described previously: volume was determined using Vernier caliper, A × B^2^ × 0.52, where A is the longest diameter of the tumor and B is the shortest diameter [12].

### 4.15. Patient Lung Sample Collection

Lung tumor tissue and normal-adjacent lung tissue from two chromate workers were obtained from the Tokushima University Hospital, Tokushima, Japan [51]. Worker 1 was a male non-smoking chromate worker (age 62) who had been exposed to chromates (Na_2_Cr_2_O_7_, K_2_Cr_2_O_7_, CrO_3_, and Cr_2_O_3_) in Hokkaido, Japan for 19 years. This worker was diagnosed with stage I (T1N0M0) squamous lung carcinoma. Worker 2 was a male non-smoking chromate worker (age 61) who had been exposed to chromates (Na_2_Cr_2_O_7_, K_2_Cr_2_O_7_, CrO_3_, and Cr_2_O_3_) in Hokkaido, Japan for 38 years. This worker was diagnosed with stage II (T2N1M0) squamous lung carcinoma.

### 4.16. Immunofluorescence

Formalin-fixed tissue slices from the chromate workers were incubated at 60 °C overnight to dissolve the paraffin. Tissues then went through a series of xylene and ethanol washes to remove the paraffin and rehydrate the tissue. Tissues were incubated with an unmasking solution for 30 min at 95 °C then washed twice with TBS (with 0.025% Triton-X). Tissues were then blocked with 10% horse serum, with 1% BSA, in TBS (with 68 0.025% Triton) and washed twice with TBS (with 0.025% Triton-X). Tissues were then incubated with primary antibodies overnight at 4 °C. Primary antibodies were diluted 1:50 in TBS with 1% BSA. Primary antibodies were then removed, and the tissues were washed twice with TBS (with 0.025% Triton-X) and re-probed with secondary antibodies at room temperature for 1 h. Secondary antibodies were then removed, and the tissues were washed three times with TBS. Slides were mounted with a cover slip and mounting solution containing DAPI. DAPI was used as a stain for the nuclei to help visualize the locations of cells and overlaid with the lipid-related protein staining. The DAPI staining was blue, FASN was green (488 nm), ACC1 was green (488 nm), pACLY was red (568 nm), and ACLY was red (568 nm). Finally, the cells were visualized and imaged using an Olympus BX53 fluorescence microscope using a 40× objective, as previously described [1].

### 4.17. Statistics

GraphPad Prism 10 (La Jolla, CA, USA) was used to determine statistical relevance. The student’s *t*-test was used to calculate *p*-values to determine the statistical significance of the differences in means. For tumor incidence, significance was determined using the chi-squared test. A *p*-value of less than 0.05 was considered significant.

## 5. Conclusions

In conclusion, we found that Cr(VI)-transformed cells have increased lipogenesis proteins and lipogenesis as compared to passage-matched control cells. This pathway change is not unique to this lung cell type and the immortalization factor for this lung cell line does not affect the results. Drug inhibition demonstrated that FASN overexpression is important for Cr(VI)-transformed cell survival and cancer properties. Lastly, we saw that pACLY, ACLY, ACC1, and FASN expressions were increased in chromate-induced human lung tumors as compared to adjacent normal lung tissue. Increased lipogenesis and associated enzymes may be a potential therapeutic target in Cr(VI)-induced lung carcinogenesis.

## Data Availability

Data are contained within the article.

## References

[1] Kim D., Dai J., Fai L.Y., Yao H., Son Y.O., Wang L., Pratheeshkumar P., Kondo K., Shi X., Zhang Z. (2015). Constitutive activation of epidermal growth factor receptor promotes tumorigenesis of Cr(VI)-transformed cells through decreased reactive oxygen species and apoptosis resistance development. J. Biol. Chem..

[2] Tsuneta Y., Ohsaki Y., Kiyonobu K., Mikami H., Abe S., Murao M. (1980). Chromium content of lungs of chromate workers with lung cancer. Thorax.

[3] Wang X., Son Y.O., Chang Q., Sun L., Hitron J.A., Budhraja A., Zhang Z., Ke Z., Chen F., Luo J. (2011). NADPH oxidase activation is required in reactive oxygen species generation and cell transformation induced by hexavalent chromium. Toxicol. Sci..

[4] Xie H., Holmes A.L., Wise S.S., Huang S., Peng C., Wise J.P. (2007). Neoplastic transformation of human bronchial cells by lead chromate particles. Am. J. Respir. Cell Mol. Biol..

[5] Clementino M., Xie J., Yang P., Li Y., Lin H.P., Fenske W.K., Tao H., Kondo K., Yang C., Wang Z. (2020). A Positive Feedback Loop Between c-Myc Upregulation, Glycolytic Shift, and Histone Acetylation Enhances Cancer Stem Cell-like Property and Tumorigenicity of Cr(VI)-transformed Cells. Toxicol. Sci..

[6] Wang L., Wise J.T.F., Zhang Z., Shi X. (2016). Progress and prospects of free radicals in metal-induced carcinogenesis. Curr. Pharmacol. Rep..

[7] Wise S.S., Elmore L.W., Holt S.E., Little J.E., Antonucci P.G., Bryant B.H., Wise J.P. (2004). Telomerase-mediated lifespan extension of human bronchial cells does not affect hexavalent chromium-induced cytotoxicity or genotoxicity. Mol. Cell Biochem..

[8] Wise S.S., Holmes A.L., Wise J.P. (2008). Hexavalent chromium-induced DNA damage and repair mechanisms. Rev. Environ. Health.

[9] Abreu P.L., Ferreira L.M.R., Alpoim M.C. (2014). Impact of hexavalent chromium on mammalian cell bioenergetics: Phenotypic changes, molecular basis and potential relevance to chromate-induced lung cancer. Biometals.

[10] Cerveira J.F., Sánchez-Aragó M., Urbano A.M., Cuezva J.M. (2014). Short-term exposure of nontumorigenic human bronchial epithelial cells to carcinogenic chromium(VI) compromises their respiratory capacity and alters their bioenergetic signature. FEBS Open Bio..

[11] Guo L., Xiao Y., Wang Y. (2013). Hexavalent chromium-induced alteration of proteomic landscape in human skin fibroblast cells. J. Proteome Res..

[12] Wise J.T.F., Wang L., Alstott M.C., Ngalame N.N.O., Wang Y., Zhang Z., Shi X. (2018). Investigating the Role of Mitochondrial Respiratory Dysfunction during Hexavalent Chromium-Induced Lung Carcinogenesis. J. Environ. Pathol. Toxicol. Oncol..

[13] Roy R.V., Pratheeshkumar P., Son Y.O., Wang L., Hitron J.A., Divya S.P., Zhang Z., Shi X. (2016). Different roles of ROS and Nrf2 in Cr(VI)-induced inflammatory responses in normal and Cr(VI)-transformed cells. Toxicol. Appl. Pharmacol..

[14] Warburg O., Wind F., Negelein E. (1927). The metabolism of tumors in the body. J. Gen. Physiol..

[15] Warburg O. (1956). On the origin of cancer cells. Science.

[16] Warburg O. (1956). On respiratory impairment in cancer cells. Science.

[17] Otto A.M. (2016). Warburg effect(s)-a biographical sketch of Otto Warburg and his impacts on tumor metabolism. Cancer Metab..

[18] Hanahan D., Weinberg R.A. (2011). Hallmarks of cancer: The next generation. Cell.

[19] Sun L., Suo C., Li S.T., Zhang H., Gao P. (2018). Metabolic reprogramming for cancer cells and their microenvironment: Beyond the Warburg Effect. Biochim. Biophys. Acta. Rev. Cancer.

[20] Santos C.R., Schulze A. (2012). Lipid metabolism in cancer. FEBS J..

[21] Menendez J.A., Lupu R. (2007). Fatty acid synthase and the lipogenic phenotype in cancer pathogenesis. Nat. Rev. Cancer.

[22] Migita T., Narita T., Nomura K., Miyagi E., Inazuka F., Matsuura M., Ushijima M., Mashima T., Seimiya H., Satoh Y. (2008). ATP citrate lyase: Activation and therapeutic implications in non-small cell lung cancer. Cancer Res..

[23] Orita H., Coulter J., Lemmon C., Tully E., Vadlamudi A., Medghalchi S.M., Kuhajda F.P., Gabrielson E. (2007). Selective inhibition of fatty acid synthase for lung cancer treatment. Clin. Cancer Res..

[24] Osugi J., Yamaura T., Muto S., Okabe N., Matsumura Y., Hoshino M., Higuchi M., Suzuki H., Gotoh M. (2015). Prognostic impact of the combination of glucose transporter 1 and ATP citrate lyase in node-negative patients with non-small lung cancer. Lung Cancer.

[25] Piyathilake C.J., Frost A.R., Manne U., Bell W.C., Weiss H., Heimburger D.C., Grizzle W.E. (2000). The expression of fatty acid synthase (FASE) is an early event in the development and progression of squamous cell carcinoma of the lung. Hum. Pathol..

[26] Zaytseva Y.Y., Harris J.W., Mitov M.I., Kim J.T., Butterfield D.A., Lee E.Y., Weiss H.L., Gao T., Evers B.M. (2015). Increased expression of fatty acid synthase provides a survival advantage to colorectal cancer cells via upregulation of cellular respiration. Oncotarget.

[27] Jin X., Zhang K.J., Guo X., Myers R., Ye Z., Zhang Z.P., Li X.F., Yang H.S., Xing J.L. (2014). Fatty acid synthesis pathway genetic variants and clinical outcome of non-small cell lung cancer patients after surgery. Asian Pac. J. Cancer Prev..

[28] Visca P., Sebastiani V., Botti C., Diodoro M.G., Lasagni R.P., Romagnoli F., Brenna A., De Joannon B.C., Donnorso R.P., Lombardi G. (2004). Fatty acid synthase (FAS) is a marker of increased risk of recurrence in lung carcinoma. Anticancer Res..

[29] Kitteringham N.R., Abdullah A., Walsh J., Randle L., Jenkins R.E., Sison R., Goldring C.E., Powell H., Sanderson C., Williams S. (2008). Proteomic analysis of Nrf2 deficient transgenic mice reveals cellular defence and lipid metabolism as primary Nrf2-dependent pathways in the liver. J. Proteom..

[30] Swinnen J.V., Brusselmans K., Verhoeven G. (2006). Increased lipogenesis in cancer cells: New players, novel targets. Curr. Opin. Clin. Nutr. Metab. Care.

[31] Little J.L., Kridel S.J. (2008). Fatty acid synthase activity in tumor cells. Subcell. Biochem..

[32] Baud S., Lepiniec L. (2009). Regulation of de novo fatty acid synthesis in maturing oilseeds of Arabidopsis. Plant Physiol. Biochem..

[33] Bu S.Y., Mashek M.T., Mashek D.G. (2009). Suppression of long-chain acyl-CoA synthetase 3 decreases hepatic de novo fatty acid synthesis through decreased transcriptional activity. J. Biol. Chem..

[34] López M., Lelliott C.J., Vidal-Puig A. (2007). Hypothalamic fatty acid metabolism: A housekeeping pathway that regulates food intake. Bioessays.

[35] Mashima T., Seimiya H., Tsuruo T. (2009). De novo fatty-acid synthesis and related pathways as molecular targets for cancer therapy. Br. J. Cancer.

[36] Suagee J.K., Corl B.A., Crisman M.V., Wearn J.G., McCutcheon L.J., Geor R.J. (2010). *De novo* fatty acid synthesis and NADPH generation in equine adipose and liver tissue. Comp. Biochem. Physiol. B Biochem. Mol. Biol..

[37] Tarun A.S., Vaughan A.M., Kappe S.H. (2009). Redefining the role of de novo fatty acid synthesis in Plasmodium parasites. Trends Parasitol..

[38] Brusselmans K., Vrolix R., Verhoeven G., Swinnen J.V. (2005). Induction of Cancer Cell Apoptosis by Flavonoids Is Associated with Their Ability to Inhibit Fatty Acid Synthase Activity. J. Biol. Chem..

[39] Corominas-Faja B., Cuyàs E., Gumuzio J., Bosch-Barrera J., Leis O., Martin Á.G., Menendez J.A. (2014). Chemical inhibition of acetyl-CoA carboxylase suppresses self-renewal growth of cancer stem cells. Oncotarget.

[40] Migita T., Okabe S., Ikeda K., Igarashi S., Sugawara S., Tomida A., Soga T., Taguchi R., Seimiya H. (2014). Inhibition of ATP citrate lyase induces triglyceride accumulation with altered fatty acid composition in cancer cells. Int. J. Cancer.

[41] Relat J., Blancafort A., Oliveras G., Cufí S., Haro D., Marrero P.F., Puig T. (2012). Different fatty acid metabolism effects of (-)-epigallocatechin-3-gallate and C75 in adenocarcinoma lung cancer. BMC Cancer.

[42] Xin M., Qiao Z., Li J., Liu J., Song S., Zhao X., Miao P., Tang T., Wang L., Liu W. (2016). miR-22 inhibits tumor growth and metastasis by targeting ATP citrate lyase: Evidence in osteosarcoma, prostate cancer, cervical cancer and lung cancer. Oncotarget.

[43] Li W., Zhang C., Du H., Huang V., Sun B., Harris J.P., Richardson Q., Shen X., Jin R., Li G. (2016). Withaferin A suppresses the up-regulation of acetyl-coA carboxylase 1 and skin tumor formation in a skin carcinogenesis mouse model. Mol. Carcinog..

[44] Yang Y.A., Han W.F., Morin P.J., Chrest F.J., Pizer E.S. (2002). Activation of fatty acid synthesis during neoplastic transformation: Role of mitogen-activated protein kinase and phosphatidylinositol 3-kinase. Exp. Cell Res..

[45] Conde E., Suarez-Gauthier A., García-García E., Lopez-Rios F., Lopez-Encuentra A., García-Lujan R., Morente M., Sanchez-Verde L., Sanchez-Cespedes M. (2007). Specific pattern of LKB1 and phospho-acetyl-CoA carboxylase protein immunostaining in human normal tissues and lung carcinomas. Hum. Pathol..

[46] Hess D., Igal R.A. (2011). Genistein downregulates de novo lipid synthesis and impairs cell proliferation in human lung cancer cells. Exp. Biol. Med..

[47] Ali A., Levantini E., Teo J.T., Goggi J., Clohessy J.G., Wu C.S., Chen P.L., Yang H., Krishnan I., Kocher O. (2018). Fatty acid synthase mediates EGFR palmitoylation in EGFR mutated non-small cell lung cancer. EMBO Mol. Med..

[48] Bollu L.R., Katreddy R.R., Blessing A.M., Pham N., Zheng B., Wu X., Weihua Z. (2015). Intracellular activation of EGFR by fatty acid synthase dependent palmitoylation. Oncotarget.

[49] Ke Y., Reddel R.R., Gerwin B.I., Miyashita M., McMenamin M., Lechner J.F., Harris C.C. (1988). Human bronchial epithelial cells with integrated SV40 virus T antigen genes retain the ability to undergo squamous differentiation. Differentiation.

[50] Willey J.C., Broussoud A., Sleemi A., Bennett W.P., Cerutti P., Harris C.C. (1991). Immortalization of normal human bronchial epithelial cells by human papillomaviruses 16 or 18. Cancer Res..

[51] Ewis A.A., Kondo K., Lee J., Tsuyuguchi M., Hashimoto M., Yokose T., Mukai K., Kodama T., Shinka T., Monden Y. (2001). Occupational cancer genetics: Infrequent ras oncogenes point mutations in lung cancer samples from chromate workers. Am. J. Ind. Med..

